# The Efficacy of Mirabegron in Medical Expulsive Therapy for Ureteral Stones: A Systematic Review and Meta-Analysis

**DOI:** 10.1155/2022/2293182

**Published:** 2022-03-24

**Authors:** Dawei Cai, Guangzhu Wei, Peishan Wu, Yongjin Huang, Xuanyan Che, Yong Zhang, Zhongbao Zhou, Guangqi Kong

**Affiliations:** ^1^Department of Urology, Beijing Luhe Hospital, Capital Medical University, No. 82 Xinhua South Road, Tongzhou District, Beijing 101149, China; ^2^Department of Urology, Beijing TianTan Hospital, Capital Medical University, No. 119 South 4th Ring West Road, Fengtai District, Beijing 100070, China

## Abstract

**Background:**

This study aimed to assess the efficacy of mirabegron (50 mg daily) as a medical expulsive therapy for ureteral stones in adults.

**Materials and Methods:**

We searched PubMed, Embase, Cochrane Library, and Web of Science from inception to July 2021 to collect the clinical trials. Two reviewers independently screened literature, extracted data, and assessed the risk of bias of included studies by using the Cochrane risk of bias tool. Review Manager 5.3 software was used for the meta-analysis.

**Results:**

A total of four studies were included, involving 398 patients: 197 patients in mirabegron group and 201 patients in control group. The meta-analysis showed that the stone expulsion rate was higher in the mirabegron group than in the control group (OR: 2.12; 95% CI: 1.33 to 3.40; *p*=0.002). Subgroup analysis identified that the stone expulsion rate of patients with stone size <5/6 mm was significantly higher than that of patients with stone size ≥5/6 mm (OR: 0.31; 95% CI: 0.13 to 0.72; *p*=0.006). But no significant difference was identified between the mirabegron group and the control group for the stone expulsion interval (MD: −1.16, 95% CI: −3.56 to 1.24; *p*=0.35). In terms of pain episodes, the mirabegron group was significantly lower than that of the control group (MD: −0.34, 95% CI: −0.50 to 0.19; *p* < 0.0001).

**Conclusions:**

The medical expulsive therapy with mirabegron had a significant effect in improving the stone expulsion rate for patients with ureteral stones, especially in those whose stone size <5/6 mm. Mirabegron had no effect on the stone expulsion interval but did decrease the pain episodes.

## 1. Introduction

Urolithiasis was a primary health problem in all countries, and its prevalence has been increasing for decades [1]. When a patient is diagnosed with ureteral stones, treatment may include observation, shock wave lithotripsy (SWL), drainage, or ureteroscopy, depending on the clinical characteristics of the stone [2]. However, as the size of the stone increased and the position of the stone changed, the possibility of spontaneous stone expulsion gradually decreased [3, 4].

If the condition of the patient did not require active treatment, the latest international guidelines recommended the use of medical expulsive therapy to increase the chance of spontaneous stone passing, and ultimately that may avoid surgical treatment [5, 6]. Multiple experiments have found *β*3 adrenergic receptors in the ureteral wall and bladder wall and reported that stimulation of these receptors can relax the ureter and bladder [7, 8]. Mirabegron, as a *β*3 adrenergic receptor agonist, is currently widely used to treat overactive bladder [9]. In the past decades, there were no meta-analyses evaluating mirabegron in medical expulsive therapy for ureteral stones in which the stone size was <5/6 mm and ≥5/6 mm. Therefore, the aim of this meta-analysis was to evaluate the efficacy of mirabegron (50 mg daily) in medical expulsive therapy for ureteral stones in adults.

## 2. Materials and Methods

### 2.1. Literature Search Strategy

This study has been reported in line with Preferred Reporting Items for Systematic Reviews and Meta-Analyses (PRISMA) guidelines [10]. However, the review protocol was not registered in any public registry. To identify published and unpublished trials, we used electronic databases including PubMed (inception to July 2021), Embase (inception to July 2021), Cochrane Library (inception to July 2021), and Web of Science (inception to July 2021) without language or date restrictions. The following keywords were used in the databases just cited: mirabegron, beta-3 adrenergic agonist, medical expulsive therapy, ureteral stones, urolithiasis, and ureteral calculi.

### 2.2. Study Selection Criteria

Studies selected for the meta-analysis met the following inclusive criteria: (1) clinical trial comparing the efficacy of mirabegron in medical expulsive therapy for ureteral stones with control; and (2) complete data available for analysis. The exclusion criteria were as follows: (1) studies without available data; (2) studies with duplicated data; (3) studies updated in subsequent publications; and (4) studies without merging analysis data.

### 2.3. Data Abstraction

Two authors independently carried out literature screening, evaluation, and data extraction, and all disagreements were discussed and decided by the third author. The extracted content included the first author, the year of publication, study area, date of study, the number of patients in each group, follow-up time, treatment, dosage, eligibility criteria, stone expulsion rate, stone expulsion interval, and pain episodes.

### 2.4. Assessment of Risk of Bias and Statistical Meta-Analysis

We used the Cochrane risk of bias tool to assess potential types of bias [11]. The risk of bias in each field will be divided into “low risk,” “unclear risk,” and “high risk” according to the actual situation [11]. The statistical analyses were completed with Review Manager 5.3 software. All the variables that were available in more than one study were synthesized. Dichotomous variables were presented as the odd risk (OR) with a 95% confidence interval (CI), whereas continuous variables were expressed as the mean difference (MD) with a 95% CI. The quantity of the statistical heterogeneity was tested by the *I*^2^ statistic. *I*^2^ ≥ 50% was regarded as the presence of heterogeneity, and then explored the source of heterogeneity; if required, the random-effects model was conducted for meta-analysis. When heterogeneity was considered to be low (*I*^2^ < 50%), a fixed-effects model was used for analysis. During the analysis, we only found that the stone expulsion interval had high heterogeneity (*I*^2^ = 66%) and, in this case, a random-effects model was adopted. Because only 2 included studies were included in this particular analysis, it was not possible to explore the source of heterogeneity.

## 3. Results

### 3.1. Study Characteristics

**Figure 1:**
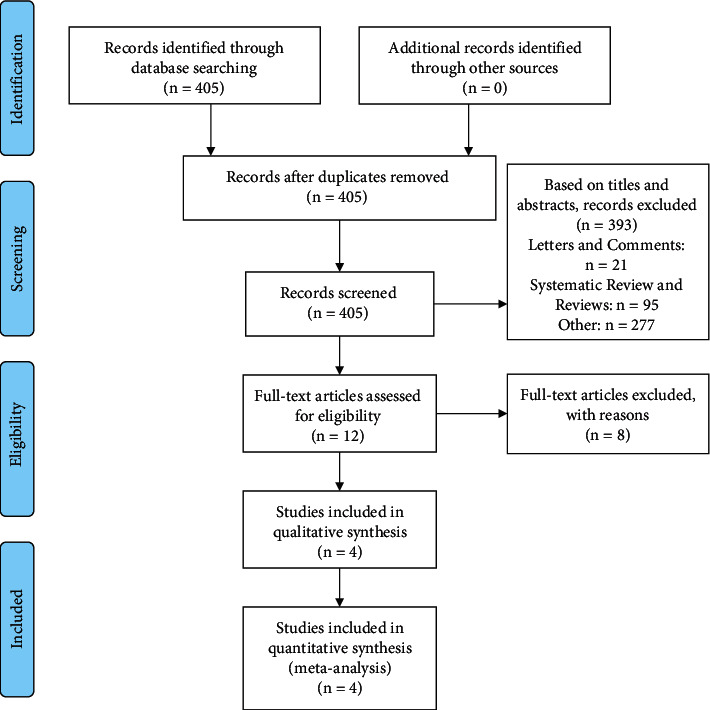
PRISMA of selection process.

**Figure 2:**
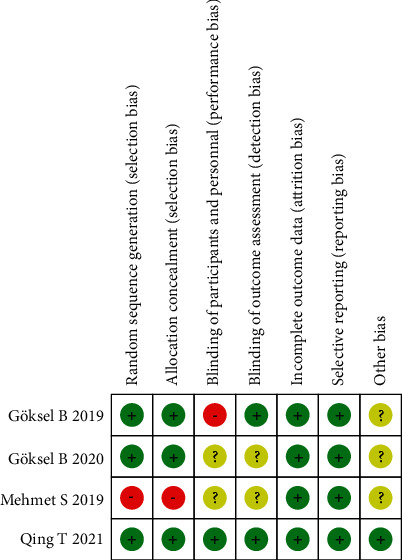
Risk of bias summary.

Following a screening of the available databases, 405 potentially relevant publications were identified. Ultimately, 4 clinical trials [12–15] were selected for the study, including 197 cases of mirabegron and 201 cases of control, to assess the effectiveness of mirabegron in medical expulsive therapy for ureteral stones. A flow diagram detailing the literature selection process is shown in Figure 1. The characteristics of these 4 trials are listed in Table 1, and the risk of bias is shown in Figure 2.

### 3.2. Stone Expulsion Rate

**Figure 3:**
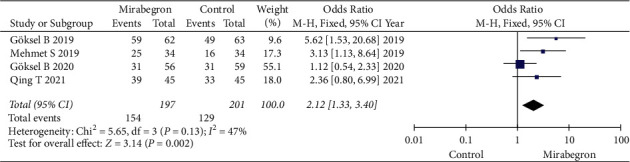
Results in stone expulsion rate between the mirabegron group and the control group.

**Figure 4:**
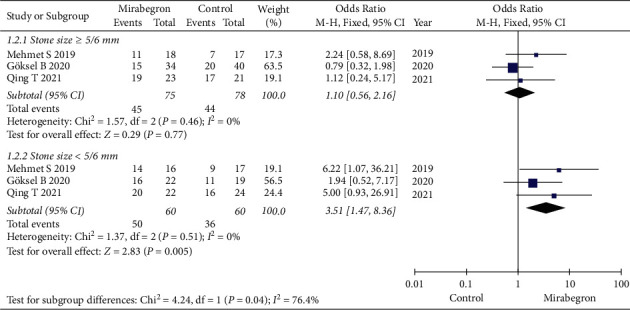
The subgroup analysis of stone expulsion rate between the mirabegron group and the control group base on stone size <5/6 mm and ≥5/6 mm.

Four articles, collecting 398 cases (197 in the mirabegron group and 201 in the control group) were involved in the research for the stone expulsion rate. The forest plots reflected an OR of 2.12 (95% CI: 1.33 to 3.40; *P*=0.002). The results revealed that the stone expulsion rate was significantly higher in the mirabegron group compared with the control group for patients with ureteral stones (Figure 3). Subgroup analysis identified that there was a marked difference between stone size <5/6 mm and stone size ≥5/6 mm in the stone expulsion rate (*P*=0.04, *I*^2^ = 76.4%) (Figure 4).

#### 3.2.1. Stone Size ≥5/6 mm

Three articles, collecting 153 cases (75 in the mirabegron group and 78 in the control group), were involved in the research for the stone expulsion rate. The forest plots reflected a OR of 1.10 (95% CI: 0.56 to 2.16, *P*=0.77), which revealed that there was no marked difference between the mirabegron group and the control group in the stone expulsion rate for patients with stone size ≥5/6 mm (Figure 4).

#### 3.2.2. Stone Size <5/6 mm

Three articles, collecting 120 cases (60 in the mirabegron group and 60 in the control group), were involved in the research for the stone expulsion rate. The forest plots reflected an OR of 3.51 (95% CI: 1.47 to 8.36, *P*=0.005), which revealed that the stone expulsion rate was significantly higher in the mirabegron group compared with the control group for patients with stone size <5/6 mm (Figure 4).

#### 3.2.3. Stone Size ≥5/6 mm vs Stone Size <5/6 mm

**Figure 5:**
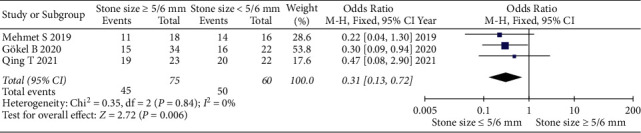
The stone expulsion rate of stone size <5/6 mm vs stone size ≥5/6 mm in the mirabegron group.

Three articles, collecting 135 cases (75 in stone size ≥5/6 mm and 60 in stone size <5/6 mm group), were involved in the research for the stone expulsion rate. The forest plots reflected an OR of 0.31 and a 95% CI of 0.13 to 0.72 (*P*=0.006), which revealed that mirabegron had a significant effect in improving the stone expulsion rate for the patients with ureteral stones, especially in the stone size <5/6 mm (Figure 5).

### 3.3. Stone Expulsion Interval

**Figure 6:**
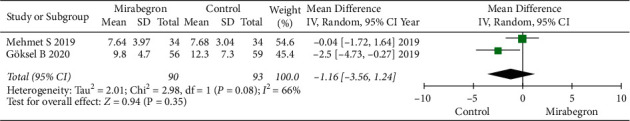
Results in stone expulsion interval between the mirabegron group and the control group.

Two articles, collecting 183 cases (90 in the mirabegron group and 93 in the control group), were involved in the research for stone expulsion interval. The forest plots reflected a MD of −1.16 and a 95% CI of −3.56 to 1.24 (*P*=0.35). The results revealed that there was no marked difference between the mirabegron group and the control group in the stone expulsion interval for patients with ureteral stones (Figure 6).

### 3.4. Pain Episodes

**Figure 7:**
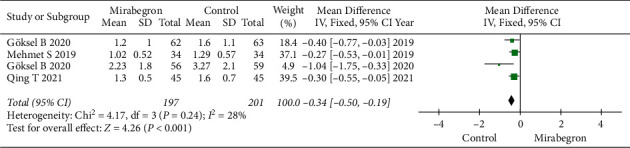
Results in pain episodes between the mirabegron group and the control group.

Four articles, collecting 398 cases (197 in the mirabegron group and 201 in the control group), were involved in the research for the pain episodes. The forest plots reflected a MD of −0.34 (95% CI: −0.50 to −0.19, *P* < 0.0001), which revealed that the mirabegron group had a less pain episodes than the control group (Figure 7).

## 4. Discussion

Ureteral stones are the most typical symptom of urolithiasis. Clinically, the spontaneous excretion rate of ureteral stones with a size of 5–10 mm was 25% to 51%, and the spontaneous excretion rate of ureteral stones smaller than 5 mm was 71% to 98% [16, 17]. Due to the role of medical expulsive therapy in alleviating stone-related symptoms and promoting stone excretion, many studies have strongly recommended this method to increase stone clearance [18, 19]. Recently, multiple clinical trials reported that mirabegron could be used as medical expulsive therapy by stimulating *β*3 adrenoceptor to relax the ureteral, which may provide a new idea for the medical expulsive therapy of ureteral stones.

The purpose of the meta-analysis was to evaluate the efficacy of mirabegron as a medical expulsive therapy for ureteral stones in adults. The analysis discovered that the stone expulsion rate was higher in the mirabegron group than in the control group (*P*=0.002). Subgroup analysis identified that the stone expulsion rate of patients with stone size <5/6 mm was significantly higher than that of patients with stone size ≥5/6 mm (*P*=0.006). But no significant difference was identified between the mirabegron group and the control group for the stone expulsion interval (*P*=0.35). In terms of pain episodes, the mirabegron group was significantly lower than that of the control group (*P* < 0.0001).

*β*3 adrenoceptor agonists have been used as a new drug for the treatment of overactive bladder, and have shown expected therapeutic effects [20]. The functional expression of *β*3 adrenoceptors in the ureter has been confirmed, and it has been found that this receptor may have an effect in ureteral peristalsis and other ureteral functions [7, 21]. One study confirmed *β*1–3 adrenoceptors were located in the smooth muscles and urothelial cells of the upper, middle, and lower ureters, where *β*2 and *β*3 adrenoceptors were especially responsible for regulating the relaxation of the ureteral wall [8]. Michel et al. reported that *β*3 adrenoceptor agonists played a relaxing role by regulating the function of the urinary tract epithelium, thus indirectly affecting muscle tone, which findings were similar to those reported in bladder [22]. Tomiyama et al. found that *β* adrenoceptor agonist significantly lowered the intraurethral pressure caused by acute ureteral obstruction and increased the urinary flow of experimental animals [23]. Shen et al. identified that the obstruction of ureteral stones led to a decrease in the number of *β*3 adrenergic receptors in the lumen, which resulted in the contraction of ureteral smooth muscle, but the number of *β*2 receptors remained stable [7]. Yalcin et al. observed that *β*-adrenergic receptor agonists inhibited the contraction of ureteral smooth muscle and dilated the ureter by reducing the frequency of peristalsis of the ureteral smooth muscle [24]. In addition, Shimamoto et al. found that the number of *β*3 receptors in the dilated distal ureter was obviously less than that in the normal ureter [7]. These studies supported our findings that *β*-adrenergic receptor agonists could be a new treatment for ureteral stones.

There were many factors that affected the spontaneous excretion of ureteral stones, mainly including the location of the stones, the size of the stones, the number of stones, mucosal edema, and ureter spasm [25]. Because of these factors, we can relieve the ureteral mucosal edema and ureteral spasm with drugs, thereby improving the spontaneous excretion of stones [26]. In our study, compared with the control group, the use of mirabegron significantly improved the stone removal rate of stones size less than 5 mm (60% vs 83%). In addition, when the stone adhered to the wall of the distal ureteral tube, it exhibited symptoms very similar to the symptoms of overactive bladder syndrome [14]. In order to alleviate such symptoms and increase the rate of stone clearance, many pharmacologic agents such as adrenergic blocker and antimuscarinics were used [27, 28]. During the clinical practice of mirabegron in treating overactive bladder, researchers also found some adverse reactions, including dry mouth, constipation, acute urinary retention, tachycardia, and urinary tract infection [20]. However, the occurrence of these adverse events was similar to that of the control group, which also showed that the patient tolerated the drug well.

There are some limitations of our study: (1) the inclusion and exclusion criteria, sample size, and experimental design of each study were different, which may lead to high heterogeneity of some outcomes; (2) there were only four studies that meet the standards; (3) some studies did not provide complete and detailed information of outcomes and complications; (4) most of the studies only provided short-term follow-up data, no mid and long-term follow-up data, and it was impossible to compare the mid and long-term effects of mirabegron in medical expulsive therapy for ureteral stones; (5) the grey literature on this topic has not been explored, which was also a limiting factor affecting this study; and (6) due to the small number of included studies, we did not analyze the source of heterogeneity in stone expulsion interval. Overall, MET with mirabegron had a significant effect in improving the stone expulsion rate for the patients with ureteral stones, especially with a stone size of <5/6 mm. Mirabegron had no effect on the stone expulsion interval but did decrease the pain episodes.

## Figures and Tables

**Table 1:** The details of each included study.

Study	Country	Study design	Treatment		Sample size		Dosage	Follow-up period	Date of study	Eligibility criteria
			Experimental	Control	Experimental	Control				
Mehmet S (2019)	Turkey	Retrospective study	Mirabegron	Control	34	34	50 mg/day	15 days	Apr 2017 to Jan 2018	Patients with stones smaller than 10 mm that were located in the intramural ureteral segment
Göksel B (2019)	Turkey	RCT	Mirabegron	Control	62	63	50 mg/day	4 weeks	Jun 2017 to Aug 2018	Patients aged 18–75 years were scheduled to undergo ureteroscopy for ureteral stones
Göksel B (2020)	Turkey	RCT	Mirabegron	Control	56	59	50 mg/day	4 weeks	NA	Patients had ureter stones in size between 4 and 10 mm
Qing T (2021)	China	RCT	Mirabegron	Control	45	45	50 mg/day	4 weeks	Dec 2019 to Nov 2020	Patients aged 18–65 years were diagnosed as distal ureteral stones ≤10 mm

RCT, randomized controlled trial; NA, not available.

## Data Availability

The datasets used and/or analyzed during the current study available from the corresponding author on reasonable request.
